# α_1_Proteinase Inhibitor Regulates CD4^+^ Lymphocyte Levels and Is Rate Limiting in HIV-1 Disease

**DOI:** 10.1371/journal.pone.0031383

**Published:** 2012-02-17

**Authors:** Cynthia L. Bristow, Mariya A. Babayeva, Michelle LaBrunda, Michael P. Mullen, Ronald Winston

**Affiliations:** 1 Weill Cornell Medical College, New York, New York, United States of America; 2 Institute for Human Genetics and Biochemistry, Vesenaz, Switzerland; 3 Holmes Regional Medical Center, Melbourne, Florida, United States of America; 4 Mount Sinai School of Medicine, New York, New York, United States of America; University of Sao Paulo, Brazil

## Abstract

**Background:**

The regulation of adult stem cell migration through human hematopoietic tissue involves the chemokine CXCL12 (SDF-1) and its receptor CXCR4 (CD184). In addition, human leukocyte elastase (HLE) plays a key role. When HLE is located on the cell surface (HLE_CS_), it acts not as a proteinase, but as a receptor for α_1_proteinase inhibitor (α_1_PI, α_1_antitrypsin, SerpinA1). Binding of α_1_PI to HLE_CS_ forms a motogenic complex. We previously demonstrated that α_1_PI deficiency attends HIV-1 disease and that α_1_PI augmentation produces increased numbers of immunocompetent circulating CD4^+^ lymphocytes. Herein we investigated the mechanism underlying the α_1_PI deficiency that attends HIV-1 infection.

**Methods and Findings:**

Active α_1_PI in HIV-1 subjects (median 17 µM, n = 35) was significantly below normal (median 36 µM, p<0.001, n = 30). In HIV-1 uninfected subjects, CD4^+^ lymphocytes were correlated with the combined factors α_1_PI, HLE_CS_^+^ lymphocytes, and CXCR4^+^ lymphocytes (r^2^ = 0.91, p<0.001, n = 30), but not CXCL12. In contrast, in HIV-1 subjects with >220 CD4 cells/µl, CD4^+^ lymphocytes were correlated solely with active α_1_PI (r^2^ = 0.93, p<0.0001, n = 26). The monoclonal anti-HIV-1 gp120 antibody 3F5 present in HIV-1 patient blood is shown to bind and inactivate human α_1_PI. Chimpanzee α_1_PI differs from human α_1_PI by a single amino acid within the 3F5-binding epitope. Unlike human α_1_PI, chimpanzee α_1_PI did not bind 3F5 or become depleted following HIV-1 challenge, consistent with the normal CD4^+^ lymphocyte levels and benign syndrome of HIV-1 infected chimpanzees. The presence of IgG-α_1_PI immune complexes correlated with decreased CD4^+^ lymphocytes in HIV-1 subjects.

**Conclusions:**

This report identifies an autoimmune component of HIV-1 disease that can be overcome therapeutically. Importantly, results identify an achievable vaccine modification with the novel objective to protect against AIDS as opposed to the current objective to protect against HIV-1 infection.

## Introduction

Hematopoiesis in humans begins with stem cell migration from fetal liver through the periphery to the stromal area of hematopoietic tissue where cells are retained, differentiated, and released as maturing progenitor cells back into the periphery. Progenitor cells subsequently migrate to functionally unique tissues such as thymus for further steps of locally-defined differentiation. Pools of stem cells and progenitor cells are retained in hematopoietic tissue throughout life providing a microenvironment for progenitor cell renewal [1]. In human adults, hematopoiesis is dependent on the chemokine receptor CXCR4 and its ligand CXCL12 with an additional role played by cell surface human leukocyte elastase (HLE_CS_), and these components are motogenic [2]–[4].

Mutations in the HLE-encoding gene *ELA2* produce periodic cycling in hematopoiesis that affect monocytes and neutrophils [5], [6]. HLE_CS_ and its granule-released counterpart (HLE_G_) are synthesized as a single molecular protein that is trafficked to the cell surface early in ontogeny and is then redirected by C-terminal processing to the granule compartment later in ontogeny [7]–[9]. Generally, HLE mutations that prevent its localization to the plasma membrane cause cyclic neutropenia, while mutations that cause exclusive localization to the plasma membrane cause severe congenital neutropenia [7]. Individuals carrying a mutation in the transcriptional repressor oncogene *GFI1* which targets *ELA2*, synthesize twice more HLE, twice fewer absolute numbers of circulating CD4^+^ and CD8^+^ lymphocytes, and 7 times more monocytic cells [10]. Thus, as opposed to the well characterized enzymatic function of HLE_G_, the primary functions of HLE_CS_ appear to be cell migration and hematopoiesis [2], [4], [11].

The physiologic ligand for HLE_CS_ is α_1_proteinase inhibitor (α_1_PI, α_1_antitrypsin, *SerpinA1*) which is synthesized in hematopoietic and hepatic tissue [12]. Evidence suggests that α_1_PI also participates in hematopoiesis, specifically thymopoiesis [13]. During thymopoiesis, a cluster of mouse genes are expressed sequentially and were previously identified to encode the T cell alloantigens, Tpre, Tthy, Tind, and Tsu [14]. The chromosomal location of these maturational markers corresponds to that of α_1_PI, and using monoclonal antibodies that discriminate these mouse maturational markers, the human equivalent was identified as α_1_PI [15].

The motogenic activities of HLE_CS_ and α_1_PI involve direct or indirect interaction with Mac-1, an α_M_β_2_ integrin [16], and members of the LDL receptor family [17], [18]. In addition, α_1_PI-HLE_CS_ complexes co-localize with the receptors CD4 and CXCR4 in polarized cells, an activity that promotes cell migration and facilitates HIV-1 binding and infectivity [11], [19], [20]. We and others have shown that pretreatment of cells with α_1_PI and other ligands of HLE_CS_ for 60 min inhibits HIV-1 binding and infectivity [21]–[23]. In contrast, we have also shown that pretreatment of cells with α_1_PI for 15 min facilitates HIV-1 binding and infectivity [19]. These opposing effects of α_1_PI may be due to the kinetics of α_1_PI–induced cell migration which begins with receptor polarization at the leading edge of the migrating cell and concludes with endocytosis of the receptor aggregate at the trailing edge of the cell [18]. After 60 min incubation with α_1_PI, the HIV-1 receptor aggregate has been internalized rendering cells temporally unable to bind to HIV-1 [22], [23]. Thus, it is likely that the principal influence of α_1_PI on HIV-1 binding and infectivity is due to its extracellular activities.

Alternative mechanisms of action have been suggested for the α_1_PI effect on HIV-1 infectivity including that it is both an inhibitor of and is a substrate of two proteinases, the HIV-1 aspartyl protease and the host proteinase furin, both of which participate in processing viral proteins [24]. Like other serine proteinase inhibitors, α_1_PI forms an irreversible, covalent-like complex with its cognate proteinase, HLE_G_ or HLE_CS_, thereby inhibiting elastolytic activity. The binding of α_1_PI to the catalytic site within HLE interrupts the electron transfer mechanism of the catalytic triad. Cleavage is not completed, and α_1_PI is not cleaved [25]. Interaction of α_1_PI with serine proteinases other than HLE, for example furin, can produce cleavage, and in this case, α_1_PI is acting as a substrate, not an inhibitor [26]. The evidence that α_1_PI is a substrate for the HIV-1 aspartyl protease implies α_1_PI competes with the protease's natural substrate (Gag-Pol) such that the decreased cleavage of Gag-Pol detected was due to substrate competition, rather than inhibition.

In addition to hepatocytes, α_1_PI is produced in bone marrow, by lymphocytic and monocytic cells in lymphoid tissue, and by the Paneth cells of the gut [27], [28]. Since α_1_PI therapy in our previous study produced increased CD4 numbers in PIzz as it did in HIV-1 patients, it can be interpreted that α_1_PI in circulation contributes to CD4 numbers [13]. Since PIzz patients have very low blood concentrations of α_1_PI and do not consistently exhibit below normal CD4 numbers, it can be interpreted that α_1_PI produced in bone marrow and lymphocytic tissue also contribute to regulating CD4 numbers.

The kinetics of T cell death and proliferation has explained, in part, the short-term depletion of the circulating pool of CD4^+^ T cells in HIV-1 infection [29]; however, an explanation for their long-term depletion is absent and involves both depletion of the circulating pool and depression of hematopoiesis [13], [30]. It was previously demonstrated that in HIV-1 disease, α_1_PI is severely deficient suggesting that insufficient α_1_PI could impede thymopoiesis [31]. In a longitudinal study, the number of immunocompetent CD4^+^ lymphocytes in HIV-1 subjects was found to increase to normal levels within 2 weeks of initiating α_1_PI augmentation therapy, and this suggests that α_1_PI participates in regulating the number of circulating CD4^+^ lymphocytes [13]. Herein is shown that in a cross-sectional study, α_1_PI correlates with the number of CD4^+^ lymphocytes in HIV-1 subjects. The presence of anti-HIV-1 IgG-α_1_PI immune complexes in HIV-1 patients is shown to cause the attendant functional deficiency in α_1_PI.

## Results

### Lymphocyte numbers are regulated by α_1_PI

In healthy individuals, the concentration of α_1_PI in serum ranges from 18–53 µM between the 5^th^ and 95^th^ percentiles, and 90–100% of this protein is in its active form as determined by inhibition of porcine pancreatic elastase [31]. To investigate the relationship between active serum α_1_PI concentration, HLE_CS_^+^, and CD4^+^ lymphocyte numbers, blood was collected from 30 healthy HIV-1 uninfected adults, 14 males and 16 females (**Table 1**). Neither serum active α_1_PI, serum CXCL12, HLE_CS_^+^ lymphocytes, or CXCR4^+^ lymphocytes, were independently correlated with CD4^+^ lymphocytes. However, using multilinear regression analysis it was found that higher CD4^+^ lymphocytes were significantly correlated with a combination of factors including higher serum active α_1_PI, lower HLE_CS_^+^ lymphocyte numbers, and higher CXCR4^+^ lymphocyte numbers (r^2^ = 0.91, p<0.001). Neither serum CXCL12 (r^2^ = 0.21) nor CCR5^+^ lymphocytes (r^2^ = 0.56) were significantly correlated with CD4^+^ lymphocytes. Using multilinear regression, absolute lymphocyte numbers (T B, and NK cells) were correlated with CXCR4^+^ lymphocytes (r^2^ = 0.90, p<0.001), but not with HLE_CS_^+^ lymphocytes, serum active α_1_PI or serum CXCL12. These results are consistent with a regulatory pathway for CD4^+^ lymphocyte numbers that includes active α_1_PIand the receptors CXCR4 and HLE_CS_.

**Table 1:** Regression analysis of CD4^+^ and absolute lymphocyte numbers in HIV-1 uninfected subjects.

	Independent Variables				
	HLE_CS_^+^ Ly	CXCR4^+^ Ly	Active α1PI	CXCL12	
	69±41 cells/µl, n = 31^a^	2033±683 cells/µl, n = 32	26±6 µM, n = 36	283±58 pM, n = 32	
Dependent Variables					Multilinear Regression^b^
**CD4^+^ Ly**1024±415 cells/µl, n = 32	p<0.001	p<0.001	p<0.001	p = 0.965	r^2^ = 0.92, p<0.001, n = 31
**Total Ly**2265±746 cells/µl, n = 35	p = 0.540	p<0.001	p = 0.812	p = 0.264	r^2^ = 0.91, p<0.001, n = 31

a Values for independent and dependent variables represent mean ± standard deviation. HLE_CS_^+^ lymphocytes and CXCR4^+^ lymphocytes in the lymphocyte gate (Ly) were quantitated using flow cytometry. Active α_1_PI and CXCL12 were quantitated in serum as described.

b Multilinear regression was performed to determine the relationship of the dependent variables to the independent variables using power of test α = 0.05. Dependent variables were considered to be significantly related to the independent variable if they contributed significantly to the multilinear regression (p<0.05). In this population sample, variables were found to have constant variance and normality.

### α_1_PI is correlated with CD4^+^ lymphocyte numbers in HIV-1 disease

In the cross-sectional study population, blood was collected from 35 HIV-1 infected adults, 33 males and 2 females. Of these 35, 26 were found to have >220 CD4 cells/µl and 9 to have <220 CD4 cells/µl at the time of blood collection. All HIV-1 infected subjects were measured for numbers of CD4^+^, CXCR4^+^, and CCR5^+^ lymphocytes, as well as serum concentrations of CXCL12 as well as total, active, and inactive α_1_PI. Of these 35 subjects, 11 had active liver disease as defined by detectable Hepatitis B or C, or elevated liver enzymes. HIV-1 infected subjects with liver disease were not different from HIV-1 infected subjects without liver disease in serum active α_1_PI (p = 0.95), serum total α_1_PI (p = 0.79), CXCR4^+^ lymphocytes (p = 0.63), or CCR5^+^ lymphocytes (p = 0.9), but exhibited significantly higher serum CXCL12 (p<0.001), HLE_CS_^+^ lymphocytes (p<0.001), and CD4^+^ lymphocytes (p = 0.04).

**Figure 1:**
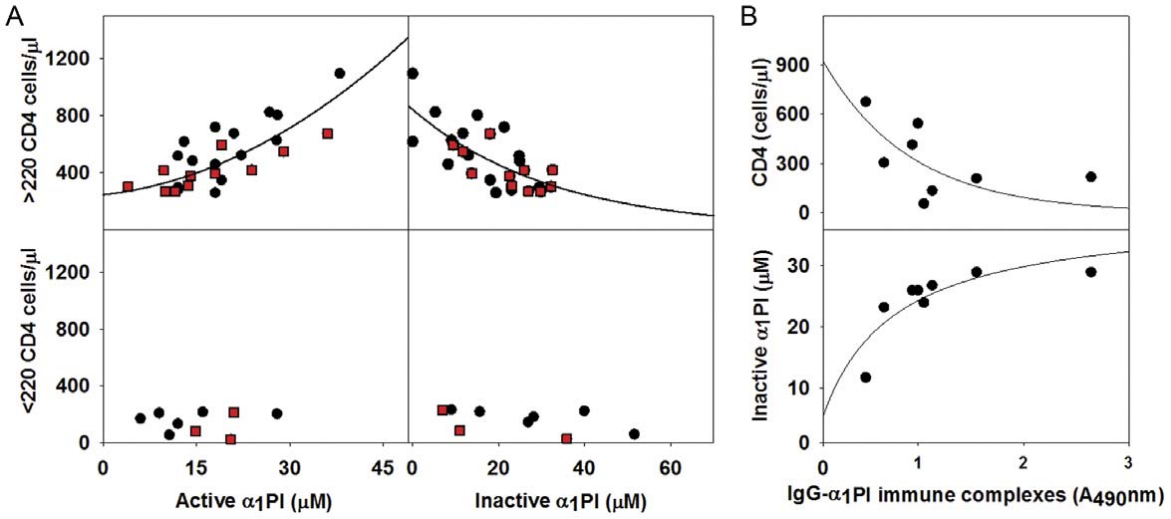
Correlation between α_1_PI, IgG-α_1_PI immune complexes, and CD4^+^ lymphocytes in HIV-1 infected subjects. (**A**) In subjects with >220 CD4 cells/µl, CD4^+^ lymphocyte levels correlate with active α_1_PI (r^2^ = 0.927, p<0.0001, n = 26). CD4^+^ lymphocyte levels also correlate with inactive α_1_PI, (r^2^ = 0.906, p<0.0001, n = 26). Subjects receiving protease inhibitor therapy are depicted by red squares. All other subjects are depicted by black circles. In the 9 subjects with <220 CD4 cells/µl, no correlation was found to exist between CD4^+^ lymphocyte levels and active α_1_PI. Non-linear regression was performed using a 3 parameter Sigmoid curve with power of test α = 0.05. In this population, all variables were found to have normality and constant variation. (**B**) In 8 of 35 subjects, IgG-α_1_PI immune complexes were detected and were correlated with CD4^+^ lymphocyte levels (r^2^ = 0.822, p = 0.05) and with inactive α_1_PI (r^2^ = 0.988, p<0.0001).

In the 26 HIV-1 infected subjects with >220 CD4 cells/µl, absolute lymphocyte counts were significantly lower in HIV-1 infected subjects than HIV-1 uninfected subjects (p = 0.03) as were CXCR4^+^ lymphocytes (p = 0.001), CD4^+^ lymphocytes (p<0.001), and active α_1_PI (p<0.001). On the other hand, total α_1_PI (p = 0.003) and inactive α_1_PI (p<0.001) were significantly higher in HIV-1 infected subjects. In these HIV-1 infected subjects, higher CD4^+^ lymphocyte numbers were correlated with higher concentration of serum active α_1_PI (r^2^ = 0.927, p<0.0001) and lower concentration of serum inactive α_1_PI (r^2^ = 0.946, p<0.0001) (**Fig. 1A**).

Regression analysis revealed a sigmoidal relationship between CD4^+^ lymphocyte numbers and active α_1_PI or inactive α_1_PI, and this is typical of many biological relationships in which a linear relationship reaches plateau at saturation (**Fig. 1A**). In contrast, in HIV-1 uninfected subjects, there was no apparent sigmoidal relationship between CD4^+^ lymphocyte numbers and active α_1_PI (r^2^ = 0.20) or inactive α_1_PI (r^2^ = 0.22), and this is due to the multiple interacting relationships between CD4^+^ lymphocytes and CXCR4^+^ lymphocytes, CXCL12 and active α_1_PI.

Of these 26 subjects, 21 were additionally measured for HLE_CS_. As in the HIV-1 uninfected population, HLE_CS_^+^ lymphocyte numbers were not independently correlated with CD4^+^ lymphocyte numbers, but in combination with higher serum active α_1_PI concentration, lower HLE_CS_^+^ lymphocytes were significantly correlated with CD4^+^ lymphocyte numbers (p = 0.01) (**Table 2**). In subjects with <220 CD4 cells/µl, there was no relationship between CD4^+^ lymphocyte numbers and active or inactive α_1_PI concentrations in serum (**Fig. 1A**), and this suggests either HIV-1 itself, or other host processes contribute to disrupting the regulation of CD4^+^ lymphocyte numbers, a question presently being addressed in a separate manuscript (unpublished observations). CD4^+^ lymphocyte numbers were not found to correlate with CXCR4^+^ lymphocyte numbers or CCR5^+^ lymphocyte numbers individually or in combination with any parameters of disease being investigated in these HIV-1 infected subjects, and this suggests that although these chemokine receptors participate during HIV-1 entry, they do not participate in the pathologic decrease in CD4^+^ lymphocytes.

**Table 2:** Regression analysis of CD4^+^ and absolute lymphocyte numbers in HIV-1 infected subjects.

	Independent Variables^a^				
	HLE_CS_^+^ Ly	CXCR4^+^ Ly	Active α1PI	CXCL12	
	93±69 cells/µln = 21	1485±531 cells/µln = 26	19±8 µMn = 26	280±53 pMn = 22	
Dependent Variables					Multilinear Regression^b^
**CD4^+^ Ly**503±210 cells/µl, n = 26	p = 0.011	p = 0.695	p<0.001	p = 0.766	r^2^ = 0.64, p = 0.003, n = 20
**Total Ly**1882±546 cells/µl, n = 26	p = 0.596	p<0.001	p = 0.16	p = 0.02	r^2^ = 0.80, p<0.001, n = 20

a Values for independent and dependent variables represent mean ± standard deviation. HLE_CS_^+^ lymphocytes and CXCR4^+^ lymphocytes in the lymphocyte gate (Ly) were quantitated using flow cytometry. Active α_1_PI and CXCL12 were quantitated in serum as described.

b Multilinear regression was performed to determine the relationship of the dependent variables to the independent variables using power of test α = 0.05. Dependent variables were considered to be significantly related to the independent variable if they contributed significantly to the multilinear regression (p<0.05). In this population sample, variables were found to have constant variance and normality.

It was previously demonstrated that early in disease, 89% HIV-1 infected subjects have detectable antibody that is reactive with α_1_PI [31]. Two monoclonal antibodies (1C1 and 3F5) which bind a conformationally determined epitope near the C5 domain of gp120 were found to also bind human α_1_PI [32]. To examine the possibility that antibodies reactive with α_1_PI might participate in the depletion of active α_1_PI, IgG-α_1_PI immune complexes were measured in all samples with sufficient residual volume. Of 22 HIV-1 infected and 21 HIV-1 uninfected individuals tested, 8 were found to be positive for detectable IgG-α_1_PI immune complexes, and all 8 were HIV-1 infected subjects. Significantly, IgG-α_1_PI immune complexes were correlated with CD4^+^ lymphocyte numbers (r^2^ = 0.822, p>0.05, n = 8) and with serum inactive α_1_PI concentration (r^2^ = 0.988, p>0.05, n = 8) (**Fig. 1B**).

### Anti-gp120 inactivates human, but not chimpanzee α_1_PI

It was hypothesized that anti-gp120 mediated depletion of active α_1_PI might be pathognomonic for HIV-1-induced AIDS. If true, this would suggest that chimpanzee α_1_PI differs from human α_1_PI since HIV-1 infected chimpanzees survive infection and regain normal numbers of CD4^+^ lymphocytes [33]. Sequence comparison revealed that other than the known polymorphisms, human α_1_PI differs from chimpanzee α_1_PI by a single amino acid (aa 385) caused by a single nucleotide change (NCBI accession numbers BT019455 and XP_522938), and this variant amino acid lies in the α_1_PI region homologous to gp120. To determine whether this sequence difference influences the binding of anti-gp120 to α_1_PI, sera were compared from 18 HIV-1 uninfected humans and 20 HIV-1 uninfected chimpanzees.

**Figure 2:**
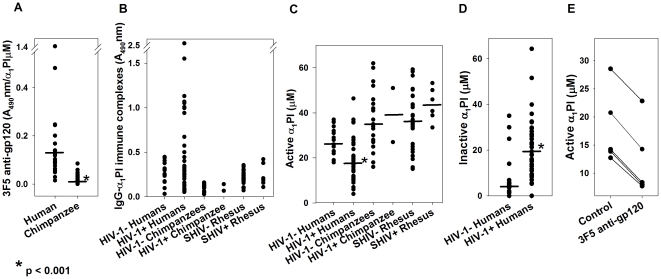
Binding of anti-gp120 to human, but not chimpanzee α_1_PI. (**A**) Monoclonal antibody 3F5 (5 µg/ml) binding to α_1_PI in sera from 18 HIV-1 uninfected humans and 20 HIV-1 uninfected chimpanzees was measured using ELISA. Antibody bound (A_490 nm_) was normalized for the active α_1_PI concentration in each specimen and is represented as A_490_nm/[α_1_PI (µM)]. Representative data from 6 measurements are depicted. Bars represent median values. Median 3F5 bound to human α_1_PI was 0.12 and to chimpanzee α_1_PI was 0.02. Negative control monoclonal antibody α70 (10 µg/ml) yielded A_490_nm = 0.02 when incubated with α_1_PI at concentrations varying between 3 µM and 540 µM. There was no difference in binding of α70 to human or chimpanzee sera (p>0.6). (**B**) IgG-α_1_PI immune complexes (A_490 nm_) were measured in sera from HIV-1 uninfected humans (n = 9), HIV-1 infected humans (n = 35), HIV-1 uninfected chimpanzees (n = 20), HIV-1 challenged chimpanzees (n = 2), rhesus monkeys pre-immunization and 2 time points post immunization (n = 12), and rhesus monkeys pre- and post-infection (n = 3). There was no significant difference in rhesus monkeys pre- and post-immunization, pre-and post-infection. Representative data of triplicate measurements are depicted. (**C**) Active α_1_PI was measured in HIV-1 uninfected humans (26 µM, n = 20), HIV-1 infected humans (18 µM, n = 35), HIV-1 uninfected chimpanzees (35 µM, n = 20), HIV-1 challenged chimpanzees (39 µM, n = 2), rhesus monkeys pre-immunization and 2 time points post immunization (36 µM, n = 12), and rhesus monkeys pre- and post-infection (43 µM, n = 3). There was no significant difference in rhesus monkeys pre- and post-immunization, pre-and post-infection. Bars represent median values. (**D**) Inactive α_1_PI was measured in HIV-1 uninfected (4 µM, n = 20) and HIV-1 infected humans (19 µM, n = 35). Bars represent median values. (**E**) Active α_1_PI levels in sera from 5 HIV-1 infected subjects after incubation with either medium (control) or with monoclonal antibody 3F5.

In contrast to chimpanzee α_1_PI, binding of both 1C1 (data not shown) and 3F5 to human α_1_PI was elevated 8- to 14-fold above background in 6 repeat measurements (p<0.001) (**Fig. 2A**). Negative control monoclonal antibody α70 which reacts with the V3-loop of gp120 failed to bind human α_1_PI consistent with previous findings [31], and there was no difference between binding of α70 to human or chimpanzee sera (p = 0.6). The markedly greater affinity for 3F5 exhibited by serum α_1_PI in two human subjects suggests that the epitope of α_1_PI recognized by 3F5 may be phenotypically determined. Even when these two subjects were omitted from the comparison, the statistical difference between binding of 3F5 to human versus chimpanzee α_1_PI was maintained (p<0.001).

None of the sera from 20 HIV-1 uninfected chimpanzees, nor sera collected from 2 chimpanzees post-HIV-1 inoculation, had evidence of detectable IgG-α_1_PI immune complexes (**Fig. 2B**). The HIV-1 inoculated chimpanzees were confirmed to be HIV-1 infected, but to have normal numbers of CD4^+^ lymphocytes (personal communication, Dr. P.N. Fultz) [34]. In addition, despite the presence of anti-gp120, there was no evidence of IgG-α_1_PI immune complexes in 12 rhesus macaques (median active α_1_PI = 36 µM) following immunization with simian/human immunodeficiency virus (SHIV 89.6) gp120 or gp140, or in 3 macaques infected with SHIV (median difference between pre- and post-immunization, A_490_nm = 0.08). Extensive *in vitro* ELISA and Western Blot analyses failed to demonstrate bi-molecular complexes between gp120 and α_1_PI which rules out the possibility that anti-gp120 association with α_1_PI was mediated by gp120. Further, the absence of detectable IgG-α_1_PI immune complexes in sera from HIV-1 infected chimpanzees suggests that gp120 and α_1_PI are not associated by aggregation in sera.

Consistent with previous evidence, the serum concentration of active α_1_PI in HIV-1 subjects (median 18 µM) was significantly below normal (median 26 µM, p<0.001) (**Fig. 2C**) and inactive α_1_PI (median 19 µM), was significantly above normal (median 4 µM, p<0.001) (**Fig. 2D**) [31]. In contrast to humans, active α_1_PI concentration in sera collected from the 2 chimpanzees post-HIV-1 inoculation (median 39 µM) were not different from normal human or chimpanzee sera (median 35 µM, p = 0.810) (**Fig. 2E**).

To determine whether α_1_PI becomes inactivated after complexing with the 3F5 anti-gp120 monoclonal antibody, 3F5 was incubated with sera samples from five healthy HIV-1 uninfected subjects. In comparison to control untreated sera, α_1_PI activity was significantly diminished to the same degree in all 3F5-treated sera (mean difference = 5.8±0.5 µM, p<0.001) (**Fig. 2E**).

To determine whether 3F5 anti-gp120 is the same antibody that produces IgG-α_1_PI immune complexes in HIV-1 infected subjects, α_1_PI activity was quantitated in 22 sera in the presence or absence of added HIV-1 virions in a blinded manner. Sera from 13 HIV-1 infected subjects with undetectable HIV RNA and >220 CD4 cells/µl, 5 of whom had detectable IgG-α_1_PI immune complexes, and 9 HIV-1 uninfected subjects were incubated with an inactivated simian immunodeficiency virus (SIV) chimera which expresses the HIV envelope (AT-2 SHIV) [35], [36]. The α_1_PI activity increased in 5 of 22 sera incubated with AT-2 SHIV, and these 5 sera were the same 5 sera from HIV-1 infected subjects that were found to have detectable IgG-α_1_PI immune complexes suggesting the α_1_PI-inactivating factor adsorbed by the virions might have been anti-gp120 (**Table 3**). In contrast, active α_1_PI was unchanged in all 9 HIV-1 uninfected sera and in all 8 of the sera from HIV-1 infected subjects lacking detectable IgG-α_1_PI immune complexes. HIV-1 infected subjects with IgG-α_1_PI immune complexes were found to have significantly lower CD4 counts (median = 210) than subjects without α_1_PI-IgG immune complexes (median = 327, p = 0.045).

**Table 3:** HIV-1 patient serum antibodies that bind the 3F5-recognized gp120 epitope are the same antibodies that bind and inactivate α_1_PI.

Subject	IgG-α_1_PI (A_490 nm_)^a^	Active α_1_PI+buffer	Active α_1_PI+SHIV	Active α_1_PI+3F5-complexed SHIV
1	0.9955	25 µM	30 µM	24 µM
2	1.0520	11 µM	19 µM	10 µM
3	1.1305	15 µM	30 µM	13 µM
4	1.5515	9 µM	17 µM	9 µM
5	2.6410	5 µM	8 µM	5 µM

a Sera were selected from HIV-1 infected subjects with undetectable HIV RNA (n = 13) or from HIV-1 uninfected subjects (n = 9). Of the 22 sera tested, only 5 exhibited increased α_1_PI activity in the presence of SHIV, and these 5 were the only specimens that were also positive for IgG-α_1_PI immune complexes (A_490_nm>0.5). Immune complexes were undetectable in the other 17 sera and none of those sera exhibited a difference in α_1_PI activity in the presence of SHIV (Mean difference = 0.7 µM±1 µM).

To demonstrate the specificity of the inactivating factor adsorbed by AT-2 SHIV, virions were pre-complexed with 3F5 monoclonal anti-gp120 prior to incubation with sera. By ELISA, it was determined that incubating AT-2 SHIV (30 µg p24) with 20 ng 3F5 in 100 µl for 45 min at 23°C results in a binding ratio of 50 ng 3F5/µg p24. After pre-complexing with 3F5, AT-2 SHIV virions were unable to remove the α_1_PI-inactivating factor and had no effect on α_1_PI activity (**Table 3**). These results demonstrate that 3F5 anti-gp120 in patient sera is the principal factor responsible for inactivating α_1_PI. Further, these results indicate that, as would be anticipated, anti-gp120 in patient sera has greater affinity for gp120 than for α_1_PI.

## Discussion

Herein is shown that active α_1_PI counterbalances HLE_CS_ in regulating CD4^+^ lymphocyte blood levels in HIV-1 infected and uninfected subjects. In contrast to HIV-1 uninfected subjects, two important differences in the HIV-1 infected population with >220 CD4 cells/µl are notable; 1) the dependence of absolute lymphocyte numbers on CXCL12, and 2) the lack of dependence of CD4^+^ lymphocytes on the number of CXCR4^+^ lymphocytes. Since there are fewer CXCR4^+^ lymphocytes in HIV-1 infected subjects (73%) and even fewer CD4^+^ lymphocytes (49%), yet no difference in serum CXCL12 or HLE_CS_^+^ lymphocytes, this suggests a flaw in the interaction between CXCR4 and CXCL12, presumably in bone marrow, that results in production of fewer CXCR4^+^ lymphocytes despite the presence of sufficient CXCL12. Alternatively, although the magnitude of CXCL12 in blood is small, CXCL12 might systemically influence the number of CXCR4^+^ lymphocytes by inducing their egress from blood into tissue. The increased CXCL12, HLE_CS_^+^ lymphocytes, and CD4^+^ lymphocytes in the presence of active liver disease in 11 HIV-1 infected patients suggests a potential regulatory axis between the liver and hematopoietic tissue that may include additional unknown factors.

In the HIV-1 uninfected population, CD4^+^ lymphocyte numbers were correlated with three factors including serum active α_1_PI, HLE_CS_^+^ and CXCR4^+^ lymphocyte numbers. Since HLE_CS_ and CXCR4 are known to participate in regulating hematopoiesis, this suggests α_1_PI, HLE_CS_ and CXCR4 regulate the number of CD4^+^ lymphocytes. Because CD4^+^ lymphocyte numbers were correlated with serum active α_1_PI concentration with such a high degree of significance in HIV-1 infected subjects with >220 CD4 cells/µl (r^2^ = 0.927, p<0.0001), it can be concluded that there is a direct regulatory link between active α_1_PI and CD4^+^ lymphocyte numbers although this doesn't show causality. Because we have previously demonstrated that α_1_PI augmentation therapy produced an increase in the number of circulating CD4^+^ lymphocytes in HIV-1 infected and uninfected subjects, it can be concluded that α_1_PI regulates the number of CD4^+^ lymphocytes in blood [13]. Since active α_1_PI regulates CD4^+^ lymphocyte numbers, yet HLE_CS_^+^ and CXCR4^+^ lymphocyte numbers appear not to contribute to regulation in the HIV-1 infected subjects, it can be concluded that active α_1_PI is the rate limiting factor for regulating CD4^+^ lymphocyte numbers in HIV-1 infected subjects. Whether the influence of α_1_PI on hematopoiesis occurs in bone marrow, the thymus, or elsewhere is currently being investigated.

Three distinct activities of α_1_PI are performed by moieties within the carboxyl terminal of the protein: (1) inhibition of soluble HLE_G_ mediated by active site residue Met^358^; disruption of this activity results in emphysema and respiratory-related infections; (2) induction of receptor polarization and cell migration mediated by α_1_PI residues ^370^ FVFLM^374^ [19]; evidence suggests that this domain stimulates cell motility and is responsible for the binding of HLE_CS_- α_1_PI complexes to members of the LDL receptor family [37], [38]; and (3) binding of α_1_PI to antibodies reactive with HIV-1 gp120 [31]; functional α_1_PI deficiency in HIV-1 disease was previously shown to be caused primarily by IgG-α_1_PI immune complex formation, not by impaired synthesis, proteolysis, or oxidation [31]. IgG-α_1_PI immune complexes were not detected in all HIV-1 infected subjects, and this may be due to the presence of immunoglobulin subclasses other than IgG, the inability to capture α_1_PI bound in immune complexes with high IgG content, or their absence in some individuals [39].

**Figure 3:**
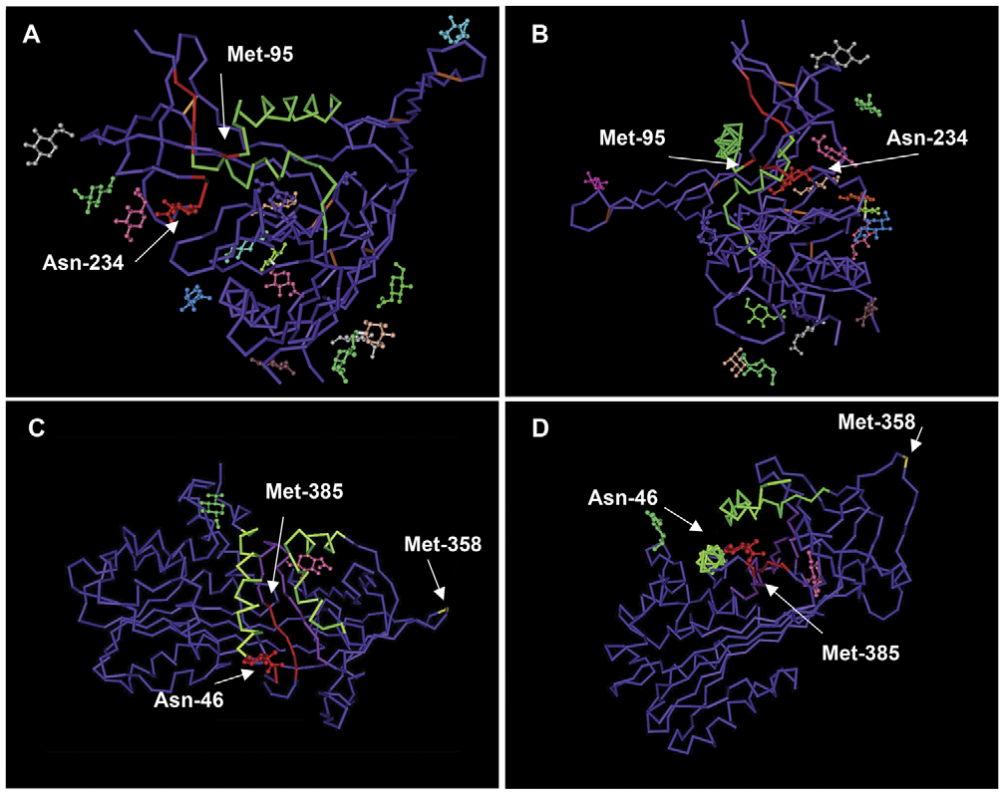
Corresponding conformation at the 3F5-recognized epitope in α_1_PI and CD4-complexed HIV-1 gp120. HIV-1 gp120 is depicted from two perspectives (**A,B**) with green representing two α-helices (aa 100–115 and 476–484). The gp120 peptide immunogen used to raise 1C1 and 3F5 (aa 471–490) is located at the C-terminus of gp120, and the linear segment ^486^YKVV^489^ is depicted in red along with Met95 and the oligosaccharide-linked segment ^234^NGT^236^, all of which are within 8 Å of the conformational epitope. The gp120-homologous domain in α_1_PI is also located at the C-terminus of the protein, and is depicted from two perspectives (**C,D**) with violet representing the antiparallel β-sheet strand at the base of the cleft (aa 369–389), and green representing the α-helices that form the mouth of the cleft (aa 27–44 and 259–277). Met-385, which distinguishes human from chimpanzee α_1_PI, is depicted in red along with the segment ^386^GKVV^389^, the oligosaccharide, and oligosaccharide-linked segment ^46^NST^48^. The HLE_G_-reactive site Met-358, is depicted in yellow for orientation. Structures for human α_1_PI (1HP7) and CD4-complexed HIV-1 gp120 (1RZJ) from the NCBI Molecular Modeling Database (MMDB) were analyzed using Cn3D software. Small carbohydrate structures, depicted in multiple colors, were associated with 1RZJ in MMDB, and the three associated with 1HP7 were added using Adobe Photoshop.

The gp120 epitope recognized by the 1C1 and 3F5 antibodies is considered to be conformation-dependent [40]. The gp120 peptide immunogen used to raise 1C1 and 3F5 (^471^GGGDMRDNWRSELYKYKVVK^490^) [41] contains both an α-helix (aa 476–484) and linear strand (aa 485–490) (**Fig. 3A,B**), but other epitope determinants of the antibodies are not known. Human α_1_PI, which also binds 1C1 and 3F5, contains (**Fig. 3C,D**), the gp120-homologous sequence (^369^PFVFLMIDQNTKSPLFMGKVV^389^) that folds to form a two-stranded antiparallel β-sheet lying at the base of a cleft (4 Å deep by 20 Å long by 5 Å wide) topped by two α-helices (aa 27–44 and 259–277) in a smaller, but similar configuration as the antigen-binding cleft of MHC (10 Å deep by 25 Å long by 10 Å wide) [42]. At the end of the first of these α-helices is the N-linked mannose-containing oligosaccharide that confers structural polymorphism to α_1_PI (N-linkage at Asn-46) [43]. In the center of the β-sheet that lies in the cleft is Met-385 which distinguishes human from chimpanzee α_1_PI (equivalent residue = Val-385). Although the function of this cleft is not known, a sequence in the center of the β-sheet formation (^370^FVFLM^374^) is homologous to the fusion domain of HIV-1 gp41 [21], and this sequence has been implicated in binding to members of the LDL receptor family and in stimulating cell migration [44] [37].

Within the α_1_PI protein, the sequence ^386^GKVV^389^ lies within 5 Å of Met-385 and Asn-46 with its N-linked oligosaccharide in a space occupying 5 Å by 5 Å by 5 Å. In the same relative orientation as in α_1_PI, the gp120 sequence ^486^YKVV^489^ lies within 5 Å of Met-95 and 8 Å of Asn-234 with its N-linked mannose-containing oligosaccharide [45] in a space of dimensions 5 Å by 5 Å by 8 Å. Significantly, the gp120 Asn-234 is invariant [46]. The N-linked oligosaccharide of α_1_PI Asn-46 confers polymorphism, and our observation that 3F5 binding to α_1_PI in 2 control sera is much greater than in the other 16 control sera (Fig. 3a) suggests this oligosaccharide may reside within the 3F5 epitope. Thus, these spatial analyses suggest that the 3F5 conformational epitope occupies a space approximately 5 Å by 5 Å by 8 Å which includes KVV, M, N, and the N-linked oligosaccharide. The dimensions of this proposed conformational epitope are consistent with previously characterized 8 Å by 7 Å antigens that contain oligosaccharide determinants [47].

Comparison of the amino acid sequences of human α_1_PI, HIV-1, HIV-2, SIV (including SIV_CPZ_), HTLV-1, and HTLV-2 reveals that all share homology with the hydrophobic core of the fusion domain of HIV-1 gp41 (LFLGFL), but only HIV-1 gp120 shares homology with the C-terminal domain of α_1_PI [31]. This observation is consistent with the species-specific differences in disease caused by the corresponding immunodeficiency viral infections. For example, neither SIV nor HTLV produce AIDS in their natural hosts [48]. SIV causes CD4 depletion in some simian species, but the progression to AIDS is disproportionately faster than in HIV-1 infection of humans. HIV-2 infection may induce AIDS, however, the frequency of AIDS incident to HIV-2 infection is 20-fold lower than for HIV-1, suggesting a variant mechanism of disease progression [49]. That antibodies reactive with α_1_PI are produced in response to a homologous sequence unique to HIV-1 suggests this immune reaction distinguishes HIV-1 infection from other retroviral infections. Importantly, a single amino acid differentiates chimpanzee α_1_PI from human α_1_PI, and this difference is in the HIV-1 gp120 homologous domain, perhaps explaining the lack of progression to AIDS in HIV-1 infected chimpanzees [33].

Molecular mimicry by viruses accommodates non-disruptive incorporation into its natural host thereby facilitating reproduction and avoiding immune clearance. Cross-species virus infection produces a situation in which molecular mimicry is less efficient thereby leading to an inflammatory response and immune recognition. Such situations can secondarily produce autoimmunity. Considering that the proteinases and proteinase inhibitors that maintain homeostasis are species-specific, the variable disease sequelae caused by SIV infection in variant monkey species could be due to the interaction of SIV or anti-SIV with a variety of homologous proteinases and proteinase inhibitors involved in homeostasis, for example, coagulation and complement proteins. Similarly, the variation in onset and course of HIV-1 and HIV-2 disease in man could be caused by differences in the interaction of HIV-1, HIV-2, anti-HIV-1, or anti-HIV-2 with various blood components involved in homeostasis. The binding of HIV-1-specific antibodies to human proteins is well-established, for example, Mac-1 [50], cardiolipin [51], inter-α-trypsin inhibitor [52], and MHC Class II [53]. In one study, 49 of 150 HIV-1 infected subjects were found to have autoantibodies reactive with conjugated fatty acids [54] Evidence presented here and elsewhere suggests that inactivation of α_1_PI by anti-gp120 is the fundamental reason CD4^+^ lymphocytes fail to be replenished and that α_1_PI augmentation therapy can overcome this autoimmune phenomenon and re-establish normal levels of CD4^+^ lymphocytes [13].

The ability of HIV-1 virions to liberate α_1_PI from anti-gp120 antibodies is not surprising since HIV-1, not α_1_PI, is the immunogen. The competitive advantage of HIV-1 for binding to anti-gp120 provides a unique method for therapeutically liberating α_1_PI by blocking autoimmune antibodies. In this manner, humans might co-exist with HIV-1, as chimpanzees do, without developing AIDS. Theoretically, modification of candidate HIV-1 vaccines so that antibodies that react with human proteins are avoided altogether provides a means to develop a vaccine that protects against AIDS as opposed to the current approach to develop a vaccine that protects against HIV-1 infection.

## Materials and Methods

### Ethics Statement

Collection of blood from research subjects was approved by the institutional review board of Cabrini Medical Center. Written informed consent was obtained from all subjects prior to participation in these studies. Residual sera from chimpanzees were purchased or approved for use by the LEMSIP IACUC.

### Human subjects

After obtaining informed consent, blood was collected from 30 HIV-1 seronegative, healthy adults, 14 males and 16 females, and from 39 HIV-1 seropositive adults attending clinic, 37 males and 2 females. Two of the HIV-1 uninfected individuals were healthy adults with the inherited version of α_1_PI deficiency (PI_ZZ_) and exhibited 41% and 42% CD4^+^ lymphocytes (reference range = 34%–58%). HIV-1 infected individuals with malignancies (n = 4) were omitted from analyses of CD4^+^ lymphocyte levels and are being evaluated separately. Of the remaining 35 HIV-1 infected individuals included in the study, 11 had evidence of active liver disease.

### Rhesus serum (Macaca mulatta)

Adult monkeys were immunized with SHIV or were infected with SHIV 89.6 as described in a previous report [55]. Residual sera from 12 immunized and 3 infected monkeys were obtained from Dr. D.C. Montefiori with approval from the Duke University Medical Center IACUC.

### Chimpanzee serum (Pan troglodytes)

Residual sera from 20 uninfected HIV-1 adult chimpanzees, 10 males and 10 females, were purchased from YERKES Regional Primate Center of Emory University. Residual sera from 2 chimpanzees collected pre- and 42 months post-HIV-1 challenge were obtained from LEMSIP of NYU Medical Center after obtaining approval from the LEMSIP IACUC [34], [56]. One chimpanzee was inoculated IV with cell-free HIV-DH12 [57]. The other chimpanzee was inoculated twice via the cervical os with HIV-infected peripheral blood cells from a chimpanzee infected with HIV LAI/IIIB and once IV with cell-free HIV-LAI/IIIB [34]. Both chimpanzees were previously confirmed to be infected and to have normal CD4^+^ lymphocyte levels [34].

### Flow cytometric analysis

Surface staining for markers on cells was performed by incubating whole blood for 15 min at 23°C with anti-CD4-FITC, anti-CXCR4-PE, anti-CCR5-PE, isotype controls (BD Biosciences, San Diego, CA). Cells were subsequently stained to detect HLE_CS_ by incubating whole blood for an additional 15 min at 23°C with rabbit anti-HLE (Biodesign, Kennebunkport, ME) or negative control rabbit IgG (Chemicon, Temecula, CA) which had been conjugated to Alexa Fluor 647 (Molecular Probes, Eugene, OR). After lysing red blood cells and washing, stained cells were fixed. At least 10,000 cells from each sample were acquired using a FACSCalibur flow cytometer. Markers on cells in the lymphocyte gate were quantitated, and CD4^+^ cells in the lymphocyte gate were compared with measurements obtained from an outside contractor to validate gating. In all cases CD4^+^ lymphocyte numbers were within 95% agreement between the two laboratories. Cell staining was analyzed using CellQuest (BD Biosciences) or FlowJo software (Tree Star, Inc., Ashland, OR).

### Quantitation of serum CXCL12, α_1_PI, α_2_M, and anti-α_1_PI

Serum CXCL12 was measured in duplicate using an ELISA kit according to the manufacturer (R&D Systems, Minneapolis, MN). Total serum α_1_PI protein was determined in 8 serial serum dilutions by ELISA using previously described methods with the modification that serum dilution buffers contained 10 mM EDTA [58]. Active and inactive fractions of α_1_PI in once-thawed sera were detected using inhibition of porcine pancreatic elastase (PPE, Sigma, St. Louis, MO) as previously described [58] with the modification that end-point, rather than kinetic, analysis was measured [58]. IgG-α_1_PI immune complexes were measured in triplicate in phosphate buffered saline pH 7.2 by incubating sera in wells of a microtiter plate pre-coated with chicken anti-human α_1_PI IgG (OEM Concepts, Toms River, NJ) as previously described [31], and captured α_1_PI-complexed IgG was detected using peroxidase-conjugated rabbit anti-human IgG (Sigma), a reagent that was confirmed to react with chimpanzee and rhesus macaque IgG. Serum from HIV-1 uninfected subjects that had been collected into tubes containing clot activating additive were excluded from immune complex analysis because of buffer incompatibility.

### Binding of anti-gp120 antibody to α_1_PI

Human or chimpanzee sera from HIV-1 uninfected subjects were diluted 1∶10 in 200 µl phosphate buffered saline containing, 5% fish gelatin and 10 mM EDTA to which was added 10 µl containing 1 µg murine monoclonal antibodies 1C1 (Repligen, Inc., Cambridge, MA) or 3F5 (hybridoma culture supernatant, 0085-P3F5-D5-F8, a generous gift from Dr. Larry Arthur, NCI-Frederick). Monoclonal 1C1 and 3F5 were raised against a peptide immunogen from the HIV-1 gp120 C5 domain (aa 471–490, GGGDMRDNWRSELYKYKVVK) [40] and bind a conformational epitope [32]. Alternatively, sera were incubated with 10 µl containing 2 µg negative control antibody Clone α70 (ICN Biochemicals, Aurora, OH) reactive with the V3 loop of HIV-1 gp120. Immune complexes that were formed by adding anti-gp120 monoclonal antibodies to human or chimpanzee sera were captured by incubating sera in wells of a microtiter plate pre-coated with chicken anti-human α_1_PI IgG (OEM Concepts). Binding was detected using horse radish peroxidase-conjugated rabbit anti-mouse IgG (Sigma) followed by substrate, orthophenylene diamine HCl.

### Inactivated virus adsorption of antibodies from α_1_PI

The AT-2-inactivated SHIV preparation used in this study was generously provided by Jeff Lifson, Julian Bess, and Larry Arthur of the AIDS Vaccine Program (SAIC-Frederick, National Cancer Institute at Frederick, Frederick, MD, USA). Chemically inactivated non-infectious virus with conformationally and functionally intact envelope glycoproteins was produced by treatment with AT-2 as described [35], [36]. The SHIV89.6 virus was produced from the CEM X 174 (T1) cell line [35]. Virus content of purified concentrated preparations was determined with an antigen capture immunoassay for capsid protein (AIDS Vaccine Program). Virus stocks were diluted in 1% BSA (Intergen, New York, NY, USA) in Dulbecco's PBS and stored as aliquots (3 µg capsid protein/ml) at −80°C until use.

To determine the adsorption of anti-gp120 antibodies from α_1_PI, AT-2 SHIV (3 µg/ml) or dilution buffer (Dulbecco's PBS +1% BSA) were added to an equal volume of serum and incubated for 30 min at 23°C prior to measuring α_1_PI activity. To demonstrate the specificity of antibody adsorption, AT-2 SHIV virions (5.5 µg/120 µl) were pre-incubated with 3F5 monoclonal anti-gp120 (10.5 µg/500 µl) or dilution buffer (500 µl) for 60 min at 23°C prior to with serum. Unbound 3F5 was removed by centrifugation of AT-2 SHIV virions at 14,000 g for two hrs, and virions were resuspended in 120 µl Tris-buffered saline, pH7.8, containing 10 mM EDTA.

### Statistical Analysis

Least squares linear and multiple linear regression were performed using SigmaPlot. Unless other stated, Measurements are presented as mean ± standard deviation for absolute lymphocyte counts, CD4^+^, CXCR4^+^, CCR5^+^, and HLE_CS_^+^ lymphocytes, as well as serum concentrations of CXCL12, total α_1_PI, active α_1_PI, inactive α_1_PI, IgG-α_1_PI immune complexes, and 3F5 anti-gp120 binding to human and chimpanzee α_1_PI. Absolute lymphocyte counts, CXCR4^+^, and CCR5^+^ lymphocytes were normally distributed, and means were compared. HLE_CS_^+^ lymphocytes, and serum concentrations of CXCL12, active α_1_PI, inactive α_1_PI, IgG-α_1_PI immune complexes, and 3F5 anti-gp120 binding to human and chimpanzee α_1_PI were not normally distributed, and medians were compared. CD4^+^ lymphocytes were normally distributed in the HIV-1 uninfected subjects, but not normally distributed in the HIV-1 infected subjects, and medians were compared. Means were compared using Student's t-test, and medians were compared using the Mann-Whitney Rank Sum Test.

## References

[1] Yahata T, Yumino S, Seng Y, Miyatake H, Uno T (2006). Clonal analysis of thymus-repopulating cells presents direct evidence for self-renewal division of human hematopoietic stem cells.. Blood.

[2] Lapidot T, Petit I (2002). Current understanding of stem cell mobilization: The roles of chemokines, proteolytic enzymes, adhesion molecules, cytokines, and stromal cells.. Exp Hematol.

[3] Tavor S, Petit I, Porozov S, Goichberg P, Avigdor A (2005). Motility, proliferation, and egress to the circulation of human AML cells are elastase dependent in NOD/SCID chimeric mice.. Blood.

[4] Cepinskas G, Sandig M, Kvietys PR (1999). PAF-induced elastase-dependent neutrophil transendothelial migration is associated with the mobilization of elastase to the neutrophil surface and localization to the migrating front.. J Cell Science.

[5] Horwitz M, Benson KF, Person RE, Aprikyan AG, Dale DC (1999). Mutations in ELA2, encoding neutrophil elastase, define a 21-day clock in cyclic haematopoiesis.. Nat Genet.

[6] Horwitz M, Benson KF, Duan Z, Li FQ, Person RE (2004). Hereditary neutropenia: dogs explain human neutrophil elastase mutations.. Trends Mol Med.

[7] Benson KF, Li FQ, Person RE, Albani D, Duan Z (2003). Mutations associated with neutropenia in dogs and humans disrupt intracellular transport of neutrophil elastase.. Nat Genet.

[8] Gullberg U, Lindmark A, Lindgren G, Persson AM, Nilsson E (1995). Carboxyl-terminal prodomain-deleted human leukocyte elastase and cathepsin G are efficiently targeted to granules and enzymatically activated in the rat basophilic/mast cell line RBL.. J Biol Chem.

[9] Garwicz D, Lennartsson A, Jacobsen SEW, Gullberg U, Lindmark A (2005). Biosynthetic profiles of neutrophil serine proteases in a human bone marrow-derived cellular myeloid differentiation model.. Haematologica.

[10] Person RE, Li FQ, Duan Z, Benson KF, Wechsler J (2003). Mutations in proto-oncogene GFI1 cause human neutropenia and target ELA2.. Nat Genet.

[11] Banda MJ, Rice AG, Griffin GL, Senior RM (1988). α1-proteinase inhibitor is a neutrophil chemoattractant after proteolytic inactivation by macrophage elastase.. J Biol Chem.

[12] Kuiperij HB, van Pel M, de Rooij KE, Hoeben RC, Fibbe WE (2009). SerpinA1 (α1-AT) is synthesized in the osteoblastic stem cell niche.. Exp Hematol.

[13] Bristow CL, Cortes J, Mukhtarzad R, Trucy M, Franklin A, Alfano M (2010). α1Antitrypsin therapy increases CD4+ lymphocytes to normal values in HIV-1 patients.. Soluble factors mediating innate immune responses to HIV infection.

[14] Owen FL, Peterman GM (1984). Neoplastic model for the differentation of a subpopulation of lymphocytes bearing.. Immunological Reviews.

[15] Bristow CL, Flood PM (1993). T cell antigen receptor immune complexes demonstrating biologic and proteolytic activity.. Int Immunol.

[16] Cai TQ, Wright SD (1996). Human leukocyte elastase is an endogenous ligand for the integrin CR3 (CD11b/CD18,Mac-1,α_M_β_2_) and modulates polymorphonuclear leukocyte adhesion.. J Exp Med.

[17] Strickland DK, Gonias SL, Argraves WS (2002). Diverse roles for the LDL receptor family.. Trends Endocrinol Metab.

[18] Cao C, Lawrence DA, Li Y, Von Amin CA, Herz J (2006). Endocytic receptor LRP together with tPA and PAI-1 coordinates Mac-1-dependent macrophage migration.. EMBO J.

[19] Bristow CL, Mercatante DR, Kole R (2003). HIV-1 preferentially binds receptors co-patched with cell surface elastase.. Blood.

[20] Bristow CL, Wolkowicz R, Trucy M, Franklin A, Di Meo F (2008). NF-κB Signaling, Elastase Localization, and Phagocytosis Differ in HIV-1 Permissive and Nonpermissive U937 Clones.. J Immunol.

[21] Bristow CL, Fiscus SA, Flood PM, Arnold RR (1995). Inhibition of HIV-1 by modification of a host membrane protease.. Int Immunol.

[22] Shapiro L, Pott GB, Ralston AH (2001). Alpha-1-antitrypsin inhibits human immunodeficiency virus type 1.. FASEB J.

[23] Munch J, Standker L, Adermann K, Schulz A, Schindler M (2007). Discovery and Optimization of a Natural HIV-1 Entry Inhibitor Targeting the gp41 Fusion Peptide.. Cell.

[24] Cordelier P, Strayer DS (2003). Mechanisms of a1-antitrypsin inhibition of cellular serine proteases and HIV-1 protease that are essential for HIV-1 morphogenesis.. Biochim Biophys Acta.

[25] Martodam RR, Liener IE (1981). The interaction of alpha 1-antitrypsin with trypsin, chymotrypsin and human leukocyte elastase as revealed by end group analysis.. Biochim Biophys Acta.

[26] Misumi Y, Oda K, Fujiwara T, Takami N, Tashiro K (1991). Functional Expression of Furin Demonstrating Its Intracellular Localization and Endoprotease Activity for Processing of Proalbumin and Complement Pro-C3*.. J Biol Chem.

[27] Winkler IG, Hendy J, Coughlin P, Horvath A, Levesque JP (2005). Serine protease inhibitors serpina1 and serpina3 are down-regulated in bone marrow during hematopoietic progenitor mobilization.. The Journal of Experimental Medicine.

[28] Molmenti EP, Perlmutter DH, Rubin DC (1993). Cell-specific expression of α_1_-antitrypsin in human intestinal epithelium.. J Clin Invest.

[29] Ribeiro RM, Mohri H, Ho DD, Perelson AS (2002). *In vivo* dynamics of T cell activation, proliferation, and death in HIV-1 infection: Why are CD4^+^ but not CD8^+^ T cells depleted?. Proc Natl Acad Sci USA.

[30] Hellerstein MK, Hoh RA, Hanley MB, Cesar D, Lee D (2003). Subpopulations of long-lived and short-lived T cells in advanced HIV-1 infection.. J Clin Invest.

[31] Bristow CL, Patel H, Arnold RR (2001). Self antigen prognostic for human immunodeficiency virus disease progression.. Clin Diagn Lab Immunol.

[32] Moore JP, Sattentau QE, Wyatt R, Sodroski J (1994). Probing the structure of the human immunodeficiency virus surface glycoprotein gp120 with a panel of monoclonal antibodies.. J Virol.

[33] Rutjens EB-J, Verschoor E, Bogers W, Koopman G, Heeney J (2003). Lentivirus infections and mechanisms of disease resistance in chimpanzees.. Front Biosci.

[34] Girard M, Mahoney J, Wei A, van der Ryst E, Muchmore E (1998). Genital infection of female chimpanzees with human immunodeficiency virus type 1.. AIDS Res Hum Retroviruses.

[35] Arthur LO, Bess JW, Sowder RC, Benveniste RES, Mann DL (1992). Cellular proteins bound to immunodeficiency viruses: Implications for pathogenesis and vaccines.. Science.

[36] Rossio JL, Esser MT, Suryanarayana K, Schneider DK, Bess JW (1998). Inactivation of human immunodeficiency virus type 1 infectivity with preservation of conformational and functional integrity of virion surface proteins.. J Virol.

[37] Joslin G, Fallon RJ, Bullock J, Adams SP, Perlmutter DH (1991). The SEC receptor recognizes a pentapeptide neodomain of alpha-1- antitrypsin-protease.. J Biol Chem.

[38] Janciauskiene S, Moraga F, Lindgren S (2001). C-terminal fragment of [alpha]1-antitrypsin activates human monocytes to a pro-inflammatory state through interactions with the CD36 scavenger receptor and LDL receptor.. Atherosclerosis.

[39] Virella G, Wohltmann H, Sagel J, Lopes-Virella MFL, Kilpatrick M (1981). Soluble immune complexes in patients with Diabetes Mellitus: Detection and pathological significance.. Diabetologia.

[40] Moore JP, Cao Y, Ho DD, Koup RA (1994). Development of the anti-gp120 antibody responses during seroconversion to human immunodeficiency virus type 1.. J Virol.

[41] Ratner L, Haseltine W, Patarca R, Livak KJ, Starcich B (1985). Complete nucleotide sequence of the AIDS virus, HTLV-III.. Nature.

[42] Bjorkman PJ, Saper MA, Samraoui B, Bennett WS, Strominger JL (1987). Structure of the human class I histocompatibility antigen, HLA-A2.. Nature.

[43] Jeppsson JO, Lilja H, Johansson M (1985). Isolation and characterization of two minor fractions of [alpha]1,-antitrypsin by high-performance liquid chromatographic chromatofocusing.. J Chromatogr A.

[44] Strickland DK, Kounnas MZ (1997). Mechanisms of Cellular Uptake of Thrombin-Antithrombin II Complexes Role of the Low-Density Lipoprotein Receptor-Related Protein as a Serpin-Enzyme Complex Receptor.. Trends in Cardiovascular Medicine.

[45] Leonard CK, Spellman MW, Riddle L, Harris RJ, Thomas JN (1990). Assignment of intrachain disulfide bonds and characterization of potential glycosylation sites of the type 1 recombinant human immunodeficiency virus envelope glycoprotein (gp120) expressed in Chinese hamster ovary cells.. J Biol Chem.

[46] Wei X, Decker JM, Wang S, Hui H, Kappes JC (2003). Antibody neutralization and escape by HIV-1.. Nature.

[47] Cygler M, Rose DR, Bundle DR (1991). Recognition of a cell-surface oligosaccharide of pathogenic Salmonella by an antibody Fab fragment.. Science.

[48] Freissmuth D, Dierich MP, Stoiber H (2003). Role of complement in the pathogeneis of SIV infection.. Front Biosci.

[49] Marlink R, Kanki P, Thior I, Travers K, Eisen G (1994). Reduced rate of disease development after HIV-2 infection as compared to HIV-1.. Science.

[50] Rubinstein DB, Farrington GK, O'Donnell C, Hartman KR, Wright DG (1999). Autoantibodies to Leukocyte [alpha]M[beta]2 Integrin Glycoproteins in HIV Infection.. Clinical Immunology.

[51] Haynes BF, Fleming J, St. Clair EW, Katinger H, Stiegler G (2005). Cardiolipin Polyspecific Autoreactivity in Two Broadly Neutralizing HIV-1 Antibodies.. Science.

[52] Koito A, Hattori T, Murakami T, Matsushita S, Maeda Y (1989). A neutralizing epitope of human immunodeficiency virus type 1 has homologous amino acid sequences with the active site of inte-α-trypsin inhibitor.. Int Immunol.

[53] Golding H, Robey FA, Gates FT, Linder W, Beining PR (1988). Identification of homologous regions in human immunodeficiency virus I gp41 and human MHC class II beta 1 domain. I. Monoclonal antibodies against the gp41-derived peptide and patients' sera react with native HLA class II antigens, suggesting a role for autoimmunity in the pathogenesis of acquired immune deficiency syndrome.. J Exp Med.

[54] Amara A, Chaugier C, Ragnaud J-M, Geffard M (1994). Circulating autoantibodies directed against conjugated fatty acids in sera of HIV-1 infected patients.. Clin Exp Immunol.

[55] Letvin NL, Montefiori DC, Yasutomi Y, Perry HC, Davies ME (1997). Potent, protective anti-HIV immune responses generated by bimodal HIV envelope DNA plus protein vaccination.. PNAS.

[56] Rodman TC, Sullivan JJ, Bai X, Winston R (1999). The human uniqueness of HIV: innate immunity and the viral Tat protein.. Hum Immunol.

[57] Girard M, van der Ryst E, Barre-Sinoussi F, Nara P, Tartaglia J (1997). Challenge of Chimpanzees Immunized with a Recombinant Canarypox-HIV-1 Virus.. Virology.

[58] Bristow CL, di Meo F, Arnold RR (1998). Specific activity of α1proteinase inhibitor and α2macroglobulin in human serum: Application to insulin-dependent diabetes mellitus.. Clin Immunol Immunopathol.

